# Direct Costs of Dengue Hospitalization in Brazil: Public and Private Health Care Systems and Use of WHO Guidelines

**DOI:** 10.1371/journal.pntd.0003104

**Published:** 2014-09-04

**Authors:** Alessandra A. Vieira Machado, Anderson Oliveira Estevan, Antonio Sales, Kelly Cristina da Silva Brabes, Júlio Croda, Fábio Juliano Negrão

**Affiliations:** 1 Faculty of Health Sciences, Federal University of Grande Dourados, Dourados, Mato Grosso do Sul, Brazil; 2 Faculty of Math, State University of Mato Grosso do Sul, Nova Andradina, Mato Grosso do Sul, Brazil; 3 Faculty of Engineering, Federal University of Grande Dourados, Dourados, Mato Grosso do Sul, Brazil; Centers for Disease Control and Prevention, United States of America

## Abstract

**Background:**

Dengue, an arboviral disease, is a public health problem in tropical and subtropical regions worldwide. In Brazil, epidemics have become increasingly important, with increases in the number of hospitalizations and the costs associated with the disease. This study aimed to describe the direct costs of hospitalized dengue cases, the financial impact of admissions and the use of blood products where current protocols for disease management were not followed.

**Methods and Results:**

To analyze the direct costs of dengue illness and platelet transfusion in Brazil based on the World Health Organization (WHO) guidelines, we conducted a retrospective cross-sectional census study on hospitalized dengue patients in the public and private Brazilian health systems in Dourados City, Mato Grosso do Sul State, Brazil. The analysis involved cases that occurred from January through December during the 2010 outbreak. In total, we examined 8,226 mandatorily reported suspected dengue cases involving 507 hospitalized patients. The final sample comprised 288 laboratory-confirmed dengue patients, who accounted for 56.8% of all hospitalized cases. The overall cost of the hospitalized dengue cases was US $210,084.30, in 2010, which corresponded to 2.5% of the gross domestic product per capita in Dourados that year. In 35.2% of cases, blood products were used in patients who did not meet the blood transfusion criteria. The overall median hospitalization cost was higher (p = 0.002) in the group that received blood products (US $1,622.40) compared with the group that did not receive blood products (US $550.20).

**Conclusion:**

The comparative costs between the public and the private health systems show that both the hospitalization of and platelet transfusion in patients who do not meet the WHO and Brazilian dengue guidelines increase the direct costs, but not the quality, of health care.

## Author Summary

The costs of dengue outbreaks and hospitalizations have recently increased. Endemic in many tropical and subtropical parts of the world, dengue outbreaks occur each year and require appropriate economic studies to determine the potential financial and public health impacts of dengue management policies. Economic literature on this topic is rare, and results are conflicting, because inconsistent assumptions are used. Health economics research specific to dengue is critical for controlling and preventing this disease. In Brazil, health care is the federal government's responsibility and is provided by the private and public health care systems; however, during an outbreak, both systems become overcrowded. Municipal governments are responsible for administering health care, with technical and financial cooperation from the government and states. The data presented here reveal the direct hospitalization costs, the private and public health care systems' costs and the impact of using the WHO guidelines on both systems. Together, these data will aid health care workers, researchers and health policy makers in financing the prevention, control and treatment of dengue fever.

## Introduction

Dengue fever (DF) is an important public health concern in tropical and subtropical regions worldwide, with approximately 100 million dengue infections and 24,000 deaths occurring annually worldwide [1], [2]. Recently, the rates of severe illness and hospitalizations related to dengue have increased, threatening public health and negatively influencing the growth of developing countries, and particularly those in Latin America [3], [4], [5].

Dengue is an arboviral disease transmitted to humans by *Aedes aegypti* [6], and the dengue virus (DENV) is a positive-strand RNA virus that belongs to the Flaviviridae family. Four genetically distinct DENVs (DENV-1, DENV-2, DENV-3 and DENV-4) cause dengue with a wide clinical spectrum of symptoms, including fever and severe dengue (SD) [7], [2]. Sequential heterotypic infections are also common in dengue-endemic areas [8].

The economic literature on the costs of DF is recent and minimal. The results are often conflicting because studies have used inconsistent assumptions. Moreover, these studies have failed to report the differences between the public and the private health care systems and the costs of hospitalization and platelet transfusion in cases in which the World Health Organization (WHO) guidelines were not followed [3], [9], [10].

In 2010, Brazil had 94,887 hospitalizations and 673 deaths due to dengue and 60.4% of worldwide reported cases of dengue illness. The State of Mato Grosso do Sul had the second highest incidence rate of dengue in Brazil, with 2,593.6 cases per 100,000 inhabitants. The prevalent dengue serotype was DENV-1, with co-circulation of DENV-2 and DENV-3 [11], [1].

We designed the present study to describe and compare the direct public and private medical costs of hospitalized dengue cases and the costs of platelet use and hospitalization with or without adherence to the criteria recommended in the WHO guidelines.

## Methods

### Study Location

**Figure 1:**
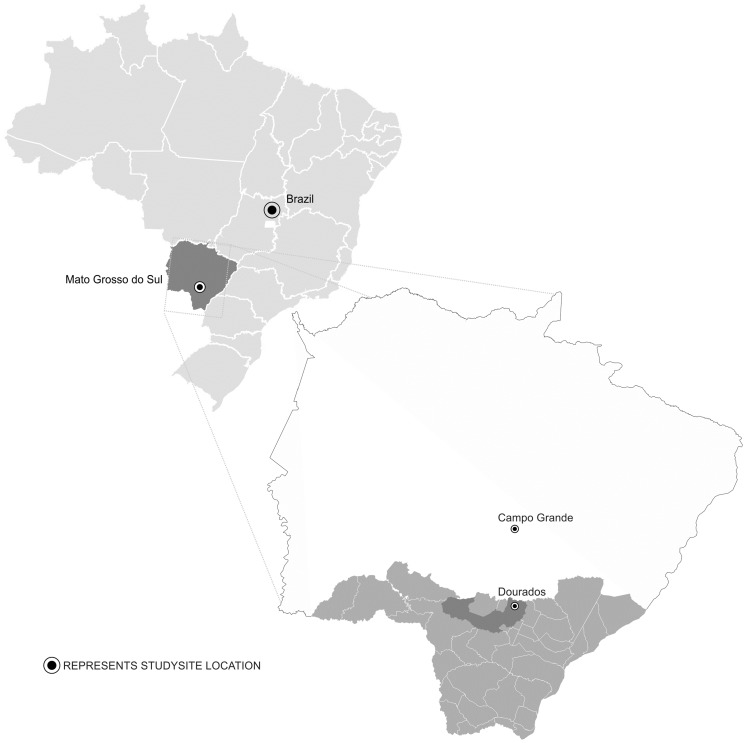
Map of Mato Grosso do Sul State, Brazil, showing Dourados in the dark area and the health care consortium network counties in the gray area.

We conducted the study in Dourados, the second largest city in the State of Mato Grosso do Sul, in the Midwest region of Brazil. This city is located 235 km from the capital, Campo Grande, at a latitude of 22°13′18.54″ South and a longitude of 54°48′23.09″ West. The estimated population of Dourados City is 196,035 inhabitants, 181,005 of whom reside in the urban area [12], [13]. Dourados, a center for public health consortia, is of economic and political importance and links 30 counties (Figure 1) [12], [13]. Preventing and controlling dengue and other epidemic outbreaks, along with all associated health care in Brazil, is the federal government's responsibility. County governments are responsible for administering the public health care system, with technical and financial assistance from the federal government and states. Moreover, hospitalization is provided by private and public health care systems.

### Hospitals' Profiles

Four hospitals were involved in the study. One was a public (university) hospital, and the other three were private. This is the unique public hospital in Dourados supported by the Unified Health System (Sistema Único de Saúde – SUS), only this hospital has pediatric and neonatal intensive care units (ICUs) and is reference for hospitalization dengue cases.

The other three hospitals treat patients with private health (PH) care, which consists of PH plans (PHPs) and payment with own resources (OR). These hospitals only have an adult ICU, and only one of hospitals has more than 50 beds.

### Study Design

This study included a retrospective survey with a cross-sectional design, and we used a bottom-up approach to determine the direct medical costs of the hospitalization of dengue cases using the Health System Agency Funding perspective [14], [15], [16]. The population consisted of all mandatorily reported dengue cases and hospital admissions from January to December 2010 in Dourados. We obtained the cases from the official database, or the National System for Reportable Diseases (Sistema Nacional de Agravos de Notificação - SINAN) [17], and from the medical records of suspected cases of dengue at the hospitals.

### Inclusion and Exclusion Criteria

We included all of the hospitalized dengue cases entered into the Information System for Disease Notification (SINAN) Dourados from January to December 2010, and we excluded dengue patients who were discharged because of a change in diagnosis.

The SINAN includes all cases reported as suspected dengue cases. Subsequently, the epidemiological surveillance team performed an investigation to confirm or exclude each case using the WHO criteria for inclusion [2]. We obtained access to the SINAN database from the Management Epidemiology and Information Municipal Health Secretariat of Dourados City and extracted the following variables: name, date of birth, sex, date and place of hospitalization. Based on these variables, we requested the medical records from the department of medical records at each institution, and the review of medical records by researchers occurred between 2012 and 2013.

In this study, we defined clinical cases of dengue as patients who were identified by physicians and who exhibited the following symptoms and clinical signs: febrile illness presenting with at least 1 clinical manifestation suggestive of dengue illness, including headache, retro-orbital pain, myalgia, joint pain, rash or any bleeding symptom [2]. For each dengue case, an NS1 ELISA, RT-PCR laboratory confirmation and serotyping tests were performed [18], [19], [20].

### WHO Dengue Classification and Management

In 2009, to identify patients at an increased risk of complications from dengue, the WHO revised its 1997 classification of DF, DHF and dengue shock syndrome (DSS), which was very rigid and limited to evaluating patients with severe clinical presentations that frequently did not meet the criteria. The new classification was based on a list of clinical warning signs suggestive of a severe disease outcome and classified dengue into dengue without warning signs (DWWS); dengue with warning signs (DWS), such as abdominal pain or tenderness, persistent vomiting, clinical fluid accumulation, mucosal bleeding, lethargy/restlessness, liver enlargement greater than 2 cm and an increase in the hematocrit concurrent with a rapid decrease in the platelet count; and severe dengue (SD) [2]. The criteria used for SD were as follows: shock, fluid accumulation with respiratory distress, severe bleeding, an AST or ALT level greater than or equal to 1,000, impaired consciousness and severe involvement of the heart and other organs [21], [22], [23].

In 2010, the Brazilian Ministry of Health continued to classify dengue cases using the 1997 WHO classification. Thus, we reclassified past dengue cases according to the new classification proposed by the WHO in 2009 using information extracted from medical and laboratory records.

The criteria for hospitalization based on the WHO recommendations are as follows: i) patients with co-existing conditions that may make dengue or its management more complicated (such as pregnancy, infancy, old age, obesity, diabetes mellitus, renal failure or chronic hemolytic disease); ii) patients with certain social circumstances (such as living alone or living far from a health facility, without reliable means of transport); and iii) patients who require emergency treatment and urgent referral due to SD [2].

Platelet transfusion is indicated when severe thrombocytopenia (platelet count <20,000 mm^3^) is present, with suspected bleeding in the central nervous system and/or major bleeding from the gastrointestinal tract (and/or the vagina in adult females) [2]. We classified the cases that did not satisfy the recommended WHO criteria as i) admission without criteria (AWC) and/or ii) platelet transfusion without criteria.

### Data Collection

For sociodemographic characterization, we considered the following variables: age, sex, race and education. We also examined clinical characteristics and outcomes by analyzing the type of hospital or intensive therapy clinic, the final disease classification, the case outcome (cure or death) and the use or lack of use of hospitalization and platelet transfusion criteria. To analyze the direct medical costs, we examined the hospitalization duration, complementary examinations, medications, medical fees, inputs and the type of health care system (public or private) [15], [24]. We obtained the costs of each hospitalization directly from the hospitals' own records.

To measure the costs of assistance, we considered 2 services: public (SUS) and private (PH) [25]. The direct medical costs included payments for hospital health care, medical services and prescriptions that were made by OR, PH and the SUS. The payments made by the SUS for hospitalized cases are based on illness type using an established price to account for hospital health care, medical services and prescriptions, except complex medical procedures and laborious assays, which are paid for separately. Therefore, the SUS transfers a fixed amount to accredited hospitals using Hospital Admission Authorization (Autorização de Internação Hospitalar - AIH), or US $135.4 per hospitalization for dengue cases [26], [27].

This amount includes the costs of daily hospitalization, medications, supplies, laboratory tests and X-rays and remains constant, regardless of the number of hospitalization days or tests required. However, additional fee is paid when computed tomography (CT); magnetic resonance imaging (MRI); or other special procedures, such as a transfusion or ICU use, are required. These additional costs may contribute to the variations in the total amount paid by the SUS to the hospitals for each case [26], [27].

We compiled the PHP and OR payment amounts from private health care companies and/or the hospitals' financial departments. To calculate the health care costs of the 4 hospitals in this study, we used the final financial data present in the medical records, which were based on specific tables. We then determined the economic value of dengue and compared it with Brazil's gross domestic product (GDP) per capita for 2010 (US $330.4) [13].

### Data Analysis

The Kolmogorov-Smirnov and Shapiro-Wilk tests showed that the data were not normally distributed (p<0.0001), so we expressed the categorical variables as proportions and the continuous variables as the median and interquartile range (IQR; 75^th^ and 25^th^ percentiles). We used the Mann-Whitney U-test or the Kruskal-Wallis test to compare medians and the chi-squared test to compare proportions, with a significance level of 95%.

We double-typed the data using EpiData version 3.1 (Lauritsen JM (Ed.), Odense, Denmark) and performed statistical analyses using SAS 9.1 (SAS Institute, Cary, NC). The values were converted from reals (R$) to US dollars (US$) by the Brazilian Central Bank on December 31, 2010 (US $1.00 = R $1,695).

We also stratified the dengue costs by cost type, financing source, sociodemographic characteristics, age group and outcomes (hospitalization and/or platelet transfusion with or without use of the WHO criteria).

### Ethics Statement

The project protocols were approved by the Committee of Ethics and Research at the Federal University of Grande Dourados (UFGD; protocol number 003/2011). During all data collection, we guarantee anonymity creating alphanumeric codes to identify each patient in the study.

## Results

**Figure 2:**
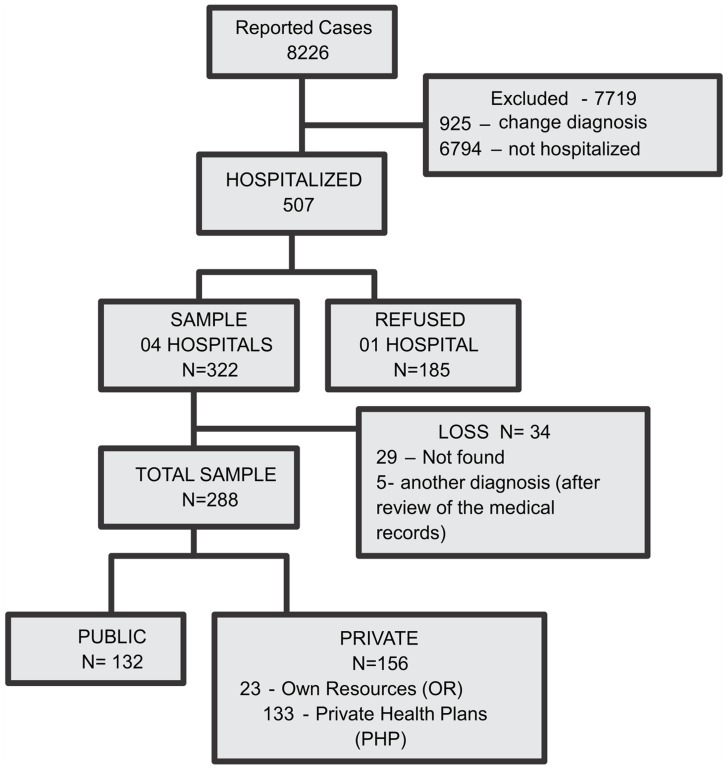
Flow chart of the selection and sampling.

In 2010, 8,226 suspected dengue cases were reported to the SINAN by Dourados, including 507 hospitalized cases. In total, 7,719 cases were excluded; 925 of these cases did not have a confirmed diagnosis of dengue, and 6,794 were not hospitalized. The final sample consisted of 288 laboratory-confirmed dengue patients, after one institute, which accounted for 36.5% of all hospitalized cases in Dourados, refused to participate. There was no significant difference in age, sex or hospitalization between patients from this hospital and those who participated. Of the 288 cases selected for analysis, 132 hospitalized cases were treated under the public health care system, and the remainder were private (Figure 2).

### Sociodemographic Characteristics and Outcomes

We stratified all sociodemographic characteristics and outcomes by health care system, and these characteristics are presented in Table 1. We observed the highest incidence of hospitalized dengue (53.5%) in the 15- to 60-year-old age group, with a median age of 39.5 years (IQR, 19 to 57 years). However, in the public health care system, children younger than 15 years old were most severely affected, which was a significantly higher rate than that in the private health care system (p<0.0001), and the education level was lower in the public health care system (p<0.0001).

**Table 1:** Characteristics of the dengue patients hospitalized in Dourados, Brazil, in 2010 (n = 288).

	Health care system				
Variable	Public (n = 132)		Private^1^ (n = 156)		X^2^
	N	%	N	%	
**Sex**					
Male	52	39.4	60	38.5	0.87
Female	80	60.6	96	61.5	
**Age group**					
<15	45	34	8	5.1	
15–60	62	47	107	68.6	<.0001
≥60	25	19	41	26.3	
**Race**					
White	82	62.1	105	67.3	
Black	5	3.8	4	2.5	0.354
Other^2^	29	22	24	14.7	
Unknown	16	12.1	24	15.5	
**Education level**					
Illiterate	4	3	1	0.7	
<9 years	34	25.7	13	8.3	
9–12 years	32	24.3	14	9	<.0001
≥12 years	12	9.1	37	23.7	
Unknown	50	37.9	91	58.3	
**Dengue classification**					
Without warning signs	56	42.4	101	64.7	0.0002
With warning signs	62	47	38	24.4	
Severe dengue	14	10.6	17	10.9	
**Comorbidities**					
Yes	48	36.4	71	45.5	
No	75	56.8	72	46.2	0.1905
Unknown	9	6.8	13	8.3	
**Type of hospitalization**					
Regular ward	117	88.7	149	95.5	
Intensive care unit (ICU)	2	1.5	1	0.7	0.0909
Regular ward and ICU	13	9.8	6	3.8	
**Presence of admission criteria**					
Yes	119	90.2	118	75.7	0.0013
No	13	9.8	38	24.3	
**Use of blood products**					
Yes	17	12.9	34	21.8	0.0483
No	115	87.1	122	78.2	
**Use of platelet transfusion criteria**					
Yes	16	88.9	17	51.5	0.0104
No	2	11.1	16	48.5	
**Case outcome**					
Recovery	131	99.2	153	98.1	0.6276
Death	1	0.8	3	1.9	

1 Included individuals with PHPs and those making payments with personal resources.

2 Mixed-race and Asiatic.

We observed the highest incidence of ‘without WS’ hospitalizations in the private health care system. However, we observed no differences in SD hospitalizations or in clinical outcomes between the public and the private health care systems.

### Cost Analysis Framework

The median cost of all reported dengue hospital admissions (n = 288) was US $259.9 (US $179.2 to US $621.2) (Table 2). The median values in the different age groups were US $201.1 (US $184.7 to US $378.7) for children younger than 15 years, US $260.7 (169.8 to 605.8) for the 15- to 60-year-old age group and US $382 (US $189.5 to US $762) for individuals older than 60 years (p = 0.003). The individuals older than 60 years stayed in the hospital for a median time of 4 days (IQR, 2 to 6 days, p = 0.003) (Table 2).

**Table 2:** Comparison of the length of the hospital stay based on age group and the type of health care system.

	Length of hospital stay (days)				
	Age group			U-test	
**Health care system**	<15	15–60	>60		**All age groups**
**Public**					
Median (IQR)	4 (5-2)	3 (4-2)	3 (4-2)	0.065	3 (5-2)
Mean (± SD)	5.3 (±5.6)	4 (±3.7)	4 (±2.6)	-	4.4 (±4.3)
**Private**					
Median (IQR)	3.5 (5-2.5)	3 (5-2)	4 (6-3)	0.2055	3 (5-2)
Mean (± SD)	3.6 (±1.3)	3.8 (±2)	4.9 (±4.6)	-	4.1 (±3.2)
**All cases** 1					
Median (IQR)	4 (5-2)	3 (5-2)	4 (6-2)	0.003	3 (5-2)
Mean (± SD)	5 (±5.2)	3.9 (±3)	4.6 (±4)	-	4.3 (±3.7)
	**Costs (US$)**				
**Public**					
Median (IQR)	193.4 (217.8-184)	169.8 (192.2-169.8)	188.7 (265.8-179.2)	<.0001	183.9(211.8-169.7)
Mean (± SD)	714 (±1469.8)	271.9 (±534.7)	303.8 (±240)	-	428.9(±954.9)
**Private**					
Median (IQR)	385.7 (857.8-321.8)	511 (924.1-259)	551.6 (1081.8-329.7)	0.6962	515.8(925.2-266)
Mean (± SD)	606.9 (±449.4)	984.5 (±2203.9)	1130.4 (±2660.8)	-	1003.5(±2274)
**All cases** 1					
Median (IQR)	201.1 (378.7-184.7)	260.7 (605.8-169.8)	382 (762.4-189.5)	0.003	259.9(621.2-179.2)
Mean (± SD)	697.9 (±1362.6)	723.1 (±1813.1)	817.3 (±2131.1)	-	740.1(±1814)

1 Public and private.

The major components of the cost analysis included the length of hospital stay, medical fees and prescriptions (medications). Moreover, we determined that the cost of dengue treatment varied greatly among patients. Table 3 compares the dengue case costs in the public and private health care systems and provides p values.

**Table 3:** Characterization of the direct medical costs by the type of health care system in Dourados City.

	Costs (US$)				
	Public (n = 132)		Private (n = 156)		U-test
	Median (IQR)	Mean (± SD)	Median (IQR)	Mean (± SD)	
Daily rate^1^	144.8 (159-135.4)	360.9 (±895.6)	157.5 (283.2-106.2)	268.2 (±472)	0.058
Imaging methods	0 (15.6-0)	10.2 (±24.2)	0 (58.5-0)	39.8 (±85.4)	0.0024
Laboratory tests	-	0	42.3 (92.3-18.7)	113.3 (±332.7)	<.0001
Consumable material	-	0	25.5 (43-15)	49.7 (±163.2)	<.0001
Medicines	-	0	84.5 (185.1-44)	280.3 (±983.6)	<.0001
Fees	34.4 (34.4-34.4)	57.6 (±81.4)	103 (188.1-59.5)	233.6 (±668.8)	<.0001
Total	183.9 (211.8-169.8)	428.7 (±954.9)	515.8 (925.1-266)	1003.5 (±2273.9)	<.0001

1 The total values include the daily rates for the regular ward and the intensive care unit and administrative fees.

When we compared the length of hospitalization and the costs of the hospitalized cases according to illness categories (WHO criteria) and the health care systems, private had significantly higher cost than public, except for SD cases (p = 0.3307) (Table 4).

**Table 4:** Comparison of the hospital stay length and costs according to the dengue classification and the type of health care system.

Length of hospital stay (days)			U-test	Total^1^	Costs (US$)		U-test	Total^1^
Dengue classification	Public	Private			Public	Private		
**WWS**								
Median (IQR)	3 (4-2)	3 (4-2)	0.8096	3(4-3)	179.2 (193.3-169.8)	389.4 (707.9-231.3)	<.0001	231.3(501.4-179.2)
Mean (± SD)	3.1 (±1.8)	3.3 (±1.9)	-	3.2(±1.9)	190.7 (±62.7)	724.5 (±1380.6)	-	534.1(±1135.4)
**WS**								
Median (IQR)	3 (6-3)	5(6-3)	0.132	4(6-3)	183.9 (212-169.8)	608.2 (1101.6-463.6)	<.0001	223.8 (612.6-169.8)
Mean (± SD)	4.6 (±3.8)	4.8 (±2.3)	-	4.7(±3.3)	351.2 (±600.8)	816.9 (±584)	-	528.1 (±633.6)
**SD**								
Median (IQR)	6 (11-3)	5 (7-4)	0.659	6(9-4)	791.3 (2701.6-204.8)	1142 (1744-601.5)	0.3307	1092 (1867.9-482.6)
Mean (± SD)	8.9 (±8.7)	7.3 (±6.7)	-	8 (±7.7)	1723.9 (±2323.6)	3078.5 (±5677.9)	-	2466.8 (±4472.5)

1 – Public and Private.

### Costs of Hospitalized Cases and Platelet Transfusion without Use of the WHO Criteria

In this study, 51 individuals were AWC, which represented 18.1% of all dengue cases (n = 288). These cases represented 11.1% (US $23,343.7) of the total hospital health care costs. 74.5% of the AWC patients (n = 38) were hospitalized in a private institution. Table 5 presents the median costs and the length of hospital stay based on the health care system type (public or private) and the use of the WHO criteria.

**Table 5:** Comparison of the hospital stay length, platelet transfusion use and costs stratified by criteria use and the type of health care system.

Hospitalization Criteria								
	With criteria		U-test	All cases	Without criteria		U-test	All cases
	Public(n = 119)	Private(n = 118)			Public(n = 13)	Private(n = 38)		
**Time (days)**								
Median	3	4	0.4004	4	2	2	0.9035	2
IQR	(5-2)	(6-3)	(6-3)		(3-1)	(3-1)	-	(3-3)
Mean	4.6	4.6	-	4.6	2.3	2.6	-	2.5
±SD	±4.5	±3.3	-	±3.9	±1.3	±2	-	±1.7
**Costs (US$)**								
Median	183.9	592.8	<.0001	265.9	174.5	262.6	0.0318	195.3
IQR	(212-169.8)	(1045-351.5)	-	(658-184)	(183.9-169.8)	(501.4-137.6)	-	(430.6-167.9)
Mean	452	1159	-	804	215.2	520.6	-	442.7
±SD	±1003	±2557.6	-	±1967.5	±120.2	±811.3	-	±713.2

Platelet transfusion								
	With criteria		U-test	All cases	Without criteria		U-test	All cases
	Public (n = 16)	Private (n = 17)			Public (n = 02)	Private (n = 16)		
**Time (days)**								
Median	6	4	0.0147	5.5	15	5.5	0.1221	6
(IQR)	(9-4)	(6-4)	-	(6.5-4)	(17-13)	(7.5-4)	-	(8-4)
Mean	6.8	5.8	-	6.3	15	6.3	-	7.2
± SD	(±2.9)	(±6.2)	-	(±4.9)	(±2.8)	(±3.7)	-	(±4.5)
**Costs (US$)**								
Median	482.5	620.8	0.0813	595.6	3165.3	1003.5	0.1449	1090.6
IQR	(207.8-1475)	(547.6-978.9)	-	(1185.7-344.8)	(2060.6-4270)	(503.7-1239)	-	(1454.3-506.7)
Mean	856.8	1811.7	-	1364	3165.3	1995.3	-	2125.3
± SD	±913.4	±4055	-	±3016.2	±1562.3	±4477.2	-	±4239.6

Platelet transfusion occurred in 17.7% (51/288) of the dengue cases, 35.2% of which (18/51) did not meet the WHO criteria. Additionally, 16 of these patients were hospitalized in the private health care system, and 2 were hospitalized in the public health care system (Table 5). The total costs were higher (p = 0.040) in the group using platelets that did not meet the WHO criteria (18/51), or US $1,090.6 (IQR, US $1,454. 3 to US $506.6), compared with the total costs in the group that was administered platelets and met the WHO criteria (n = 33), or US $595.6 (IQR, US $1,185.7 to US $344.8). However, when we separately analyzed these parameters based on health insurance (public or private), there was no statistically significant difference (Table 5).

A comparison of the length of the hospital stay and costs stratified by classification (DWWS, DWS and SD) and the type of health care system showed significant differences (Table 6), including between SD and DWS (p<.0001) and between SD and DWWS (p = 0.0002). However, when we compared DWS and DWWS, there was no statistically significant difference (p = 0.6447).

**Table 6:** Comparison of the length of the hospital stay and costs stratified by classification and the type of health care system.

	Costs (US$)			P Value^2^			
Health care system	Classification						
Public	DWWS	DWS	SD	DWWS vs DWS	SD vs DWS	SD vs DWWS	All^3^
Median	179.2	183.9	791.3	0.4128	0.0033	0.003	0.0062
(IQR)	(194-170)	(212-170)	(2701-205)	-	-	-	-
Mean	190.7	351.2	1723.9	-	-	-	-
(± SD)	(±63)	(±601)	(±2324)	-	-	-	-
**Private**							
Median	390	608	1141	0.0005	0.0007	0.1247	0.0377
(IQR)	708-232	1102-464	1744-602	-	-	-	-
Mean (± SD)	724.5	816.9	3078.5	-	-	-	-
(± SD)	±1381	±584	±5678	-	-	-	-
**All cases** 1				-	-	-	-
Median	232	224	1092	0.6447	<.0001	0.0002	<.0001
(IQR)	502-179	613-170	1868-483	-	-	-	-
Mean	534.1	528.1	2466.7	-	-	-	-
(± SD)	±1136	±634	±4473	-	-	-	-

	Length of hospital stay (days)			P Value			
	Classification						
	DWWS	DWS	SD	DWWS vs DWS	DWS vs SD	DWWS vs SD	All^3^
**Public**							
Median (IQR)	3 (4-2)	3 (6-3)	6 (6-3)	0.0679	0.0797	0.0152	0.0377
Mean (± SD)	3.2 (±1.8)	4.5 (±3.8)	8.9 (±8.7)				-
**Private**							
Median (IQR)	3 (4-2)	5 (6-3)	5 (7-4)	0.0006	0.9595	<.0001	<.0001
Mean (± SD)	3.3 (±2)	4.8 (2.3)	7.3 (±6.7)	-	-	-	-
**All cases**							
Median (IQR)	3 (4-2)	4 (6-3)	6 (9-4)	0.0004	0.0188	<.0001	<.0001
Mean (± SD)	3.2 (±1.9)	4.7 (±3.3)	8 (±7.5)	-	-	-	-

1 Public and private.

2 Calculated using the Mann-Whitney test.

3 Calculated using the Kruskal-Wallis test.

## Discussion

In this study, we determined that the direct medical costs related to dengue equaled 2.5% of the public domestic product per capita of Dourados in 2010, totaling US $210,084.3. Additionally, the median cost of hospitalizations (US $259.9) was equivalent to 78.7% of the median monthly GDP (US $330.4) of the city population studied [13].

The cost analyses indicated that the cost in the private sector was 280% higher compared with the cost in the public sector. This difference is likely due to the fact that the SUS transfers a fixed amount to accredited hospitals using AIH (US $135.4 per dengue case hospitalization) [27]. However, this amount does not represent the real cost to the SUS of a patient with dengue and demonstrates underfunding and inadequate transfer by the SUS.

Created in 1988 by the Brazilian Federal Constitution, the SUS is based on the principles of universality and equality, without any conditions, and guarantees free access to health care for approximately 190 million Brazilians [28], [26]. However, in epidemic years, the demand is often higher than the availability of health care services. Therefore, the SUS is unable to meet the demand, which forces patients to use private institutions [29]. Previous studies have indicated that the population that uses the SUS has a low level of education, fewer financial resources and a low sociodemographic profile [30], [31], [32]. In our study, we assumed that the poorest individuals are the least studied and most need the SUS. The prevention programs conducted by the PH sector were introduced very recently, and measures to prevent DF are nonexistent [28], [33].

We determined that the direct medical costs of the hospitalized cases in this study (US $259.9) were lower compared with costs in studies examining all of Brazil (US $381) [34], [4]. This difference is likely due to the fact that the authors of those studies excluded the SUS financial data and used the amounts paid by the Brazilian PHPs, whose table values are far greater than those of the SUS. However, if we consider only the private sector amounts, the costs calculated in our study were higher (US $515.8).

To our knowledge, no studies have compared the quality of the clinical management of DF regarding hospitalization criteria. However, studies of other diseases have indicated that approximately 30 to 40% of patients do not receive care according to scientific evidence and suggested guidelines. Moreover, approximately 20 to 25% of the care is not needed or is potentially harmful. Furthermore, patients who are hospitalized unnecessarily are exposed to inherent risks, such as iatrogenic or infectious diseases, which increase health care costs and the risk of death [35], [36], [37], [38].

Blood components are expensive and potentially dangerous and have a short expiration date, and their availability is often limited [39], [10]. In our study, the frequency of platelet use without meeting the recommended clinical criteria (n = 18) was similar to that reported in other recent studies [40], [41]. However, we determined that the median cost of hospitalization among patients using platelets without meeting the recommended criteria (US $1,090.6) was 83.1% higher compared with that among patients who received blood components based on the WHO protocol (US $595.6). This difference may be due to the fact that most cases occurred in the private sector, which has higher costs. However, we observed a significant difference (p = 0.002) between the groups that used (US $1,622.4) and did not use (US $550.2) blood products.

The clinical management of patients with DWS and DWWS is different according to the WHO classification, as DWS requires more imaging methods, laboratory tests and medicines depending on how serious the case is [2]. However, our study showed no significant difference between the cases of DWWS and DWS (p = 0.6447), perhaps due to non-adherence to the revised WHO guideline recommendations or other reasons not available in this study.

Our study was limited by using a secondary database. We could not evaluate indirect medical and nonmedical costs, expenditures on prevention and vector control or family income and financial impacts, and we did not determine why physicians did not follow the WHO recommendations. Furthermore, we assume the possibility of selection bias due to the refusal of 1 hospital to participate in the study (n = 185).

The new WHO guidelines better classify the severity of dengue cases [42], [43], and our study demonstrated that the use of WHO recommendations may result in savings by reducing both unnecessary hospitalizations and the use of blood products. Therefore, training needs to be offered to health care professionals to improve adherence to the revised WHO guidelines.
